# Maturation of Spinal Motor Neurons Derived from Human Embryonic Stem Cells

**DOI:** 10.1371/journal.pone.0040154

**Published:** 2012-07-03

**Authors:** Tomonori Takazawa, Gist F. Croft, Mackenzie W. Amoroso, Lorenz Studer, Hynek Wichterle, Amy B. MacDermott

**Affiliations:** 1 Department of Physiology and Cellular Biophysics, Columbia University, New York, New York, United States of America; 2 Department of Neuroscience, Columbia University, New York, New York, United States of America; 3 Department of Pathology, Columbia University, New York, New York, United States of America; 4 Department of Neurology, Columbia University, New York, New York, United States of America; 5 Center for Motor Neuron Biology and Disease, Columbia University, New York, New York, United States of America; 6 Columbia Stem Cell Initiative, Columbia University, New York, New York, United States of America; 7 Project A.L.S./Jenifer Estess Laboratory for Stem Cell Research, New York, New York, United States of America; 8 Centre for Stem Cell Biology, Sloan-Kettering Institute for Cancer Research, New York, New York, United States of America; Emory University, United States of America

## Abstract

Our understanding of motor neuron biology in humans is derived mainly from investigation of human postmortem tissue and more indirectly from live animal models such as rodents. Thus generation of motor neurons from human embryonic stem cells and human induced pluripotent stem cells is an important new approach to model motor neuron function. To be useful models of human motor neuron function, cells generated *in vitro* should develop mature properties that are the hallmarks of motor neurons *in vivo* such as elaborated neuronal processes and mature electrophysiological characteristics. Here we have investigated changes in morphological and electrophysiological properties associated with maturation of neurons differentiated from human embryonic stem cells expressing GFP driven by a motor neuron specific reporter (*Hb9*::GFP) in culture. We observed maturation in cellular morphology seen as more complex neurite outgrowth and increased soma area over time. Electrophysiological changes included decreasing input resistance and increasing action potential firing frequency over 13 days *in vitro*. Furthermore, these human embryonic stem cell derived motor neurons acquired two physiological characteristics that are thought to underpin motor neuron integrated function in motor circuits; spike frequency adaptation and rebound action potential firing. These findings show that human embryonic stem cell derived motor neurons develop functional characteristics typical of spinal motor neurons *in vivo* and suggest that they are a relevant and useful platform for studying motor neuron development and function and for modeling motor neuron diseases.

## Introduction

Motor neurons are the final connecting link between the central nervous system and skeletal muscles. They are typically large neurons with extensive dendritic fields, primarily located in the ventral horn of the spinal cord. In humans, these neurons are essentially inaccessible for study. Therefore, most of our understanding of motor neuron development and function is based on studies in a variety of mammalian model systems such as cats and rodents, extensively reviewed in [1]. Recent demonstration that human embryonic stem (hES) cells can be induced to become motor neurons (hESMNs) [2], [3], [4] has made it possible to have reliable and direct access to human motor neurons for studies of development, function and pathology.

There are several hallmarks of mammalian motor neuron maturation to which maturation of hESMN can be compared. Most noticeably, as motor neurons develop, their soma size increases and they grow morphologically more complex [5], [6]. Membrane properties also change developmentally with a decrease in input resistance, increasingly hyperpolarized resting membrane potentials and appearance of a repetitive firing response to a sustained depolarizing stimulus [5], [7], [8], [9].

Many motor neurons show additional characteristic membrane properties. For example, many motor neurons have spike frequency adaptation (SFA), defined as an increase in inter spike interval (ISI) during a repetitive firing response to a steady depolarizing current [10], [11], [12]. SFA patterns of firing during the first few seconds of repetitive action potential activity have been thought to contribute to optimal development of sustained muscle contraction and thus to smooth muscle movements [13]. There is also evidence that activity dependent modulation of SFA can occur, especially to SFA that develops over seconds [14], raising the possibility that SFA may contribute to motor neuron function in a dynamic way. Another physiological feature of some motor neurons is a post inhibitory rebound depolarization that can drive action potential firing called rebound action potentials (RAP) [15], [16]. RAP is a bursting discharge pattern that may contribute to rhythmic bursting and interact with central pattern generated rhythmic firing [17].

A variety of motor neuron-like characteristics have been demonstrated in stem cell-derived motor neurons. When mouse and human stem cells are differentiated into motor neurons [2], [3], [4], both express the motor neuron specific transcription factor HB9 and when these neurons are transplanted *in vivo,* both have the ability to extend axons in the host ventral root towards target muscles [2], [18]. Furthermore, mouse ES cell-derived motor neurons show some characteristic maturation-associated changes in membrane properties of motor neurons such as hyperpolarization of resting membrane potential and decreased input resistance [12]. However, several motor neuron properties that may contribute to regulated spike firing behavior have not been studied including SFA and RAP. These two properties, while not unique to motor neurons and not observed in all motor neurons, are broadly expressed in motor neuron populations.

Here we have examined whether hESMNs maturing *in vitro* develop characteristic motor neuron properties consistent with function in motor neuronal circuits including SFA and RAP. To accomplish this, hESMNs differentiated from stem cells and expressing GFP driven by a motor neuron specific reporter (*Hb9*::GFP) [2], [19] were subjected to morphological and electrophysiological analysis by whole-cell patch clamp. We found progressive maturation in morphological and electrophysiological properties of hESMNs with time *in vitro*.

## Results

### Morphological Maturation of hESMNs in Vitro

**Figure 1:**
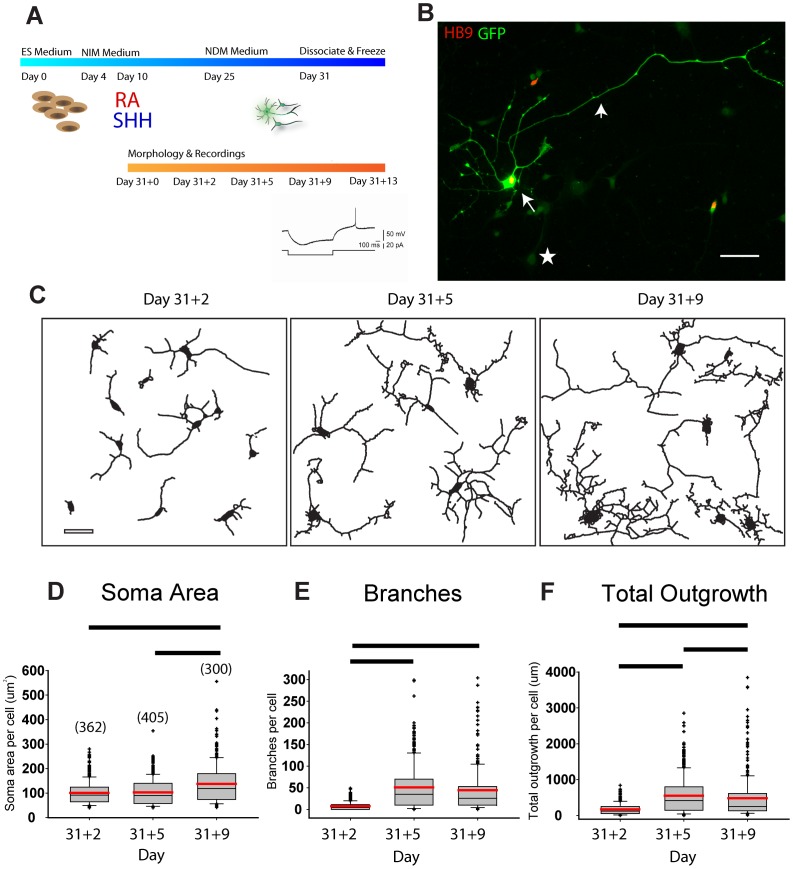
Human ES-derived motor neurons show increasing morphological complexity as they mature *in vitro*. (A) Top, schematic of ES cell directed differentiation to motor neurons shows timing of addition of the inductive cues retinoic acid (RA), and sonic hedgehog (SHH). Bottom, timing of morphometric and electrophysiological analyses. (B) Representative image of day 31+5 hESMN showing mature neuronal morphology and co-expression of GFP with motor neuron marker HB9. GFP intensity distinguished hESMN cell bodies (arrow, ∼65,000 gray levels (g.l.)), neurites (arrowhead, ∼18,000 g.l.), and cytoplasmic GFP background in non-MNs (star, ∼800 g.l.). Scale bar 50 µm. (C) Representative camera lucida (Metamorph) neurite traces from 5 randomly chosen (every 8^th^) image fields at day 31+2, 31+5, and 31+9 show increasing neurite size and complexity. Scale bar 40 µm. (D-F) Soma area, branches, total neurite outgrowth and processes (not shown) were quantified (number of cells analyzed at each timepoint shown in brackets in D), median (grey line), mean (red line), 25–75 percentile (grey box), 10–90 percentiles (whisker bars), all outliers (+) are shown for each day from which measurements were made. The values for each morphometric parameter on each day were distributed non-normally (Shapiro-Wilk test, P<0.05) and Kruskal-Wallis One Way Analysis of Variance on Ranks showed significant changes in (D) cell soma area (H = 43.885, 2 d.f., P<0.001), (E) complexity or branches/cell H = 309.245, 2 d.f., P<0.001), (F) total neurite outgrowth (H = 161.287, 2 d.f., P<0.001), and (not shown) number of primary neurites (median, 25^th^–75^th^ percentile: day 33: 3, 2–5; day 36: 6, 5–9; day 40: 8, 6–12, H = 442.555, 2 d.f., P<0.001). All significant *post hoc* pairwise comparisons, Dunn’s Method, are shown by black bars on graphs. and all pairwise comparisons for primary neurite number were significant, P<0.05.

One measure of maturation of hESMNs *in vitro* was documented by quantifying cell morphometry. Motor neurons were differentiated according to previously published protocols [3], [20] with minor modifications (Fig. 1A; see Materials and Methods). Because only a minority of differentiated cells were motor neurons, we utilized a motor neuron reporter hES cell line, where GFP expression was driven by the motor neuron specific promoter of the transcription factor HB9 [19]. GFP was useful for measurement of morphological features and allowed identification of motor neurons in cultures prior to electrophysiological recording. Embryoid bodies (EBs) began to express HB9 protein and *Hb9:*:GFP at day 25 (data not shown). EBs were dissociated to single cells on day 31 then cryopreserved for subsequent thaw and use in morphological and electrophysiological studies. Days *in vitro* (DIV) are indicated as day 31 plus the number of days in culture after being thawed.

To confirm the fidelity of GFP reporter expression within the HB9^+^ populations of neurons, cells from a day 31+5 coverslip were immunostained for both GFP and HB9 (Fig. 1B). Automated cell scoring accurately identified cells positive for either marker based on very high intensity staining (Fig. 1B). Using the criteria described in Materials and Methods, we found that 87.8% of GFP^+^ cells (n = 250 cells) were HB9^+^, similar to the 66% reported previously for this cell line [19]. Thus, the *Hb9*::GFP reporter could be used to identify hESMNs.

Morphological changes, including soma size, branching and neurite outgrowth, began shortly after hESMNs were plated. Changes in cellular morphology were monitored from day 31+0 to 31+9 *in vitro*. Camera lucida tracing of representative cells at 3 different days in culture are shown in Figure 1C. An increase in the extent and complexity of neurite outgrowth is apparent by comparing morphology at day 31+2, 31+5 and 31+9 (Fig. 1C). Cell body area, number of branches and total neurite outgrowth increased significantly during the observation period (Fig. 1D–F) as did the number of primary neurites or processes per cell (data not shown). These data establish a timeline for morphological maturation that can be correlated with physiological changes.

### Changes in Passive and Active Membrane Properties

**Figure 2:**
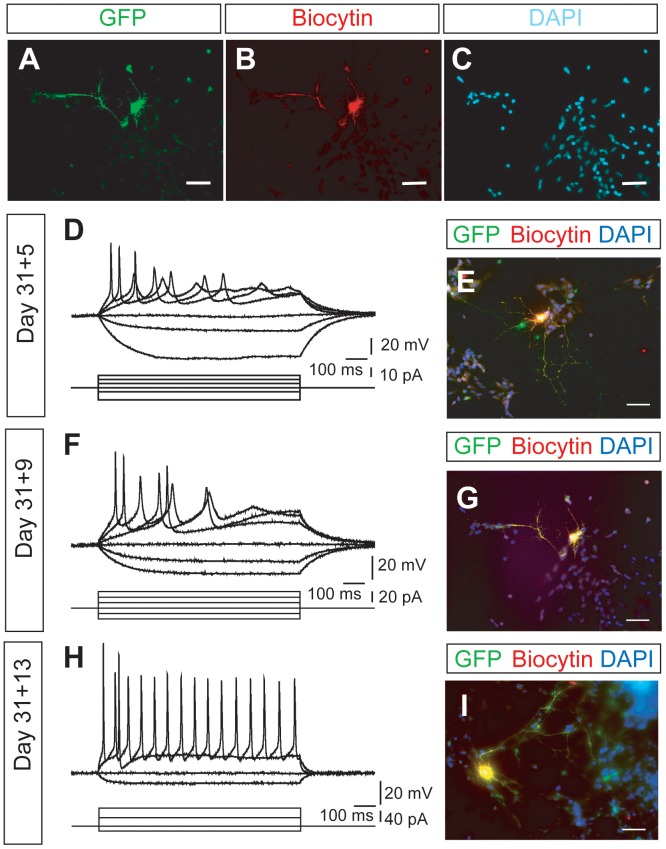
Representative morphology and membrane potential responses to current step injection in hESMNs at 3 different times *in vitro*. Imaging of cells fixed after patch-clamp recordings indicate that recorded cells express the *Hb9*::GFP reporter transgene (A-C,E,G,and I). Voltage responses and imaging in the same rows are taken from same neurons. The neurons for A-C are same as that shown in F and G. D,F,H show examples of voltage responses to current steps recorded from 3 neurons current-clamped at −58 mV, −60 mV, and −55 mV, respectively. Bottom traces in D,F, and H show injected currents. Scale bars in images are 50 μm.

The electrophysiological maturation of hESMNs over time *in vitro* was investigated by recording passive and active membrane properties from GFP expressing neurons. To confirm that recordings were accurately made on GFP-expressing motor neurons, some of the cells were loaded with biocytin during recording. They were then fixed and stained for both biocytin and GFP. Double labeling (e.g. Fig. 2A–C, E, G, I), confirmed that the neurons under study were hESMNs.

Examples of membrane potential changes in response to depolarizing and hyperpolarizing current steps together with a biocytin, GFP, DAPI triple stained image of the recorded hESMNs are shown in Figure 2D–I for 3 different hESMNs at 31+5, 31+9 and 31+13 DIV. In these examples, membrane potential responses to injected currents are clearly different among the three cells, even though all three neurons fired action potentials with stronger depolarizing stimuli. Because some of the action potentials occurring later in the trains became poorly defined, we set a criterion for identification of action potentials. Specifically, an action potential was operationally defined as having a threshold or inflection point just prior to the rapidly rising phase and a peak that overshoots 0 mV.

**Figure 3:**
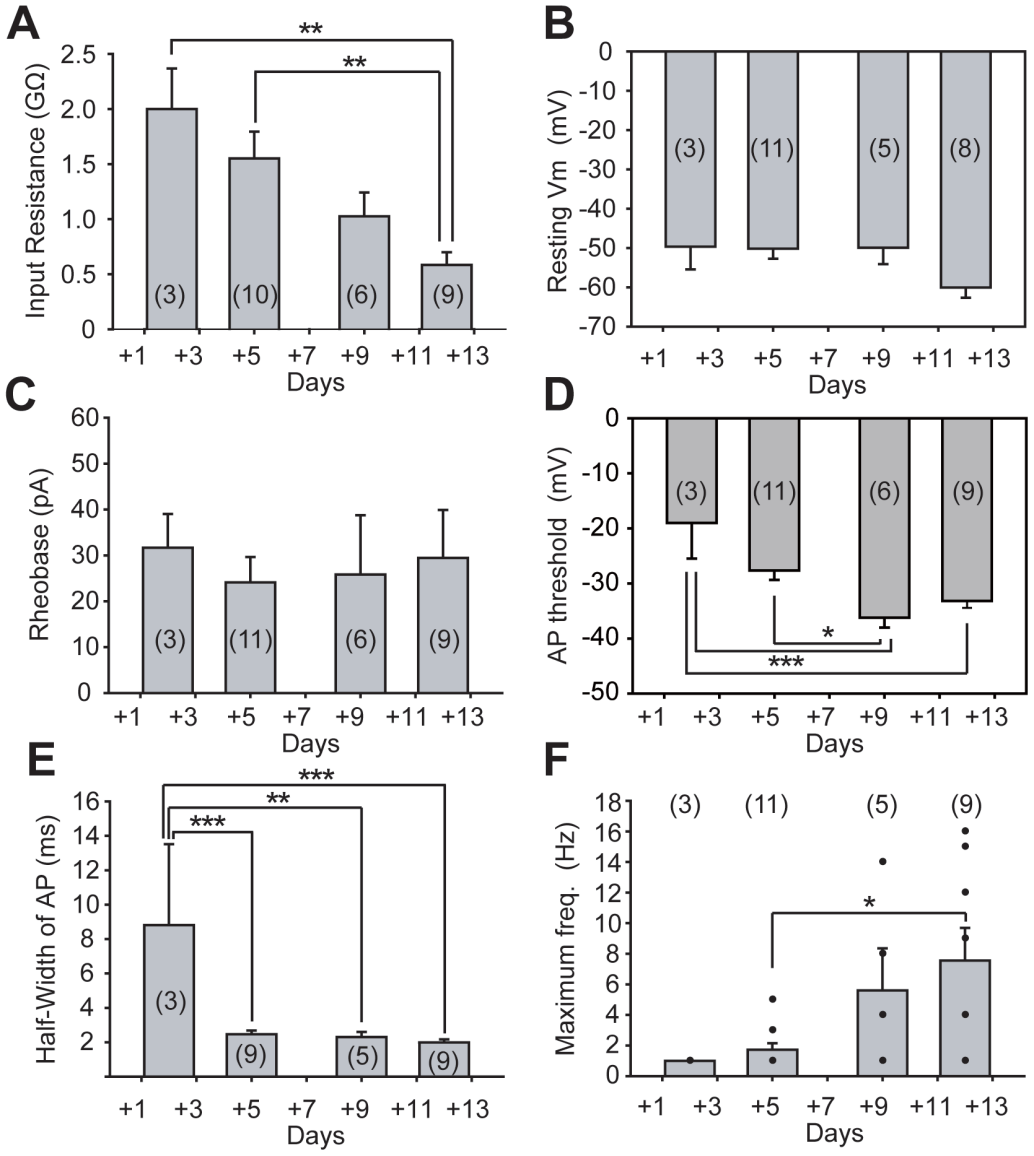
Developmental changes in intrinsic membrane properties of hESMNs. (A) Input resistance decreased over days *in vitro* (n  = 28, P<0.01, one-way ANOVA). ∗∗ P<0.01, Tukey’s *post hoc* test. (B) Resting membrane potential and (C) rheobase did not change (n  = 27 and 29, respectively). Positive current steps were injected in 5 pA increments to distinguish small differences in rheobase among individual neurons. (D) Half-width of action potentials (APs),, changed over time *in vitro* (n  = 26, P<0.001, one-way ANOVA). ∗∗ P<0.01, ∗∗∗ P<0.001, Tukey’s *post hoc* test. (E) Maximum frequency of APs after current injection increased over time *in vitro* (n  = 28, P<0.05, one-way ANOVA). ∗P<0.05, Tukey’s *post hoc* test. Dots shows frequency values for individual neurons. The numbers in parentheses indicate the number of neurons used for analysis taken from 22 dishes in total. In all panels, the first bar represents data from 31+2 DIV, 2^nd^ bar is 31+4 DIV, 3^rd^ bar is 31+8/31+9 DIV and 4^th^ bar is 31+12/31+13 DIV.

One of the clear changes in membrane properties observed as the hESMNs matured in culture was a decrease in input resistance (Fig. 2D,F,H) revealed by smaller changes in membrane potential elicited by hyperpolarizing current injections. These membrane properties are compared quantitatively over time *in vitro* in Figure 3A. Input resistance decreased with increasing DIV (Fig. 3A, n  = 28, P<0.01, one-way ANOVA), suggesting an increase in the total number of channels contributing to leak conductance at resting membrane potential. There was no significant change in resting membrane potential over the time frame of *in vitro* maturation tested here (Fig. 3B).

In all except one of the hESMNs tested (n = 28/29), at least one action potential could be evoked by depolarizing current. The one exception was a day 31+2 neuron that had been grown only 2 DIV after plating, the earliest of the days tested. Action potentials were evoked by injecting depolarizing current from resting membrane potential. Rheobase, the minimum current step required to evoke action potentials, did not change with days *in vitro* (Fig. 3C). This lack of change of rheobase in the face of decreasing input resistance predicts an accompanying hyperpolarizing change in voltage threshold. Figure 3D shows that voltage threshold changes significantly from −19 mV to −33 mV between 31+2 and 31+12/31+13 DIV (n = 29, P<0.001, one-way ANOVA). The mean duration of action potentials (i.e. half-width of action potentials) also decreased with DIV (Fig. 3E, n = 25, P<0.01, one-way ANOVA), a typical change that accompanies maturation.

Because motor neurons develop the ability to fire repetitively as they mature [9], we investigated whether a similar change occurred as the hESMNs matured in culture. None of the neurons at the youngest age tested (31+2 DIV) could produce more than a single action potential during 1 sec depolarizing current steps. However, hESMNs acquired the ability to fire a train of action potentials over time *in vitro* (Fig. 2D, 2F and 2H). Maximum frequency of action potential firing in response to depolarizing current pulses increased with DIV (Fig. 3F, n  = 28, P<0.05, one-way ANOVA), although the individual values in the same age groups showed considerable variation. Some neurons at the oldest ages tested (31+12/31+13 DIV, 3 out of 9 neurons) still produced only a single action potential, even in response to a large depolarizing current step injection (>140 pA). Overall, however, these results suggest maturation in electrophysiological function of hESMNs with time in culture that is consistent with our morphological data.

### Other Motor Neuron-like Electrophysiological Properties were Observed in hESMNs

**Figure 4:**
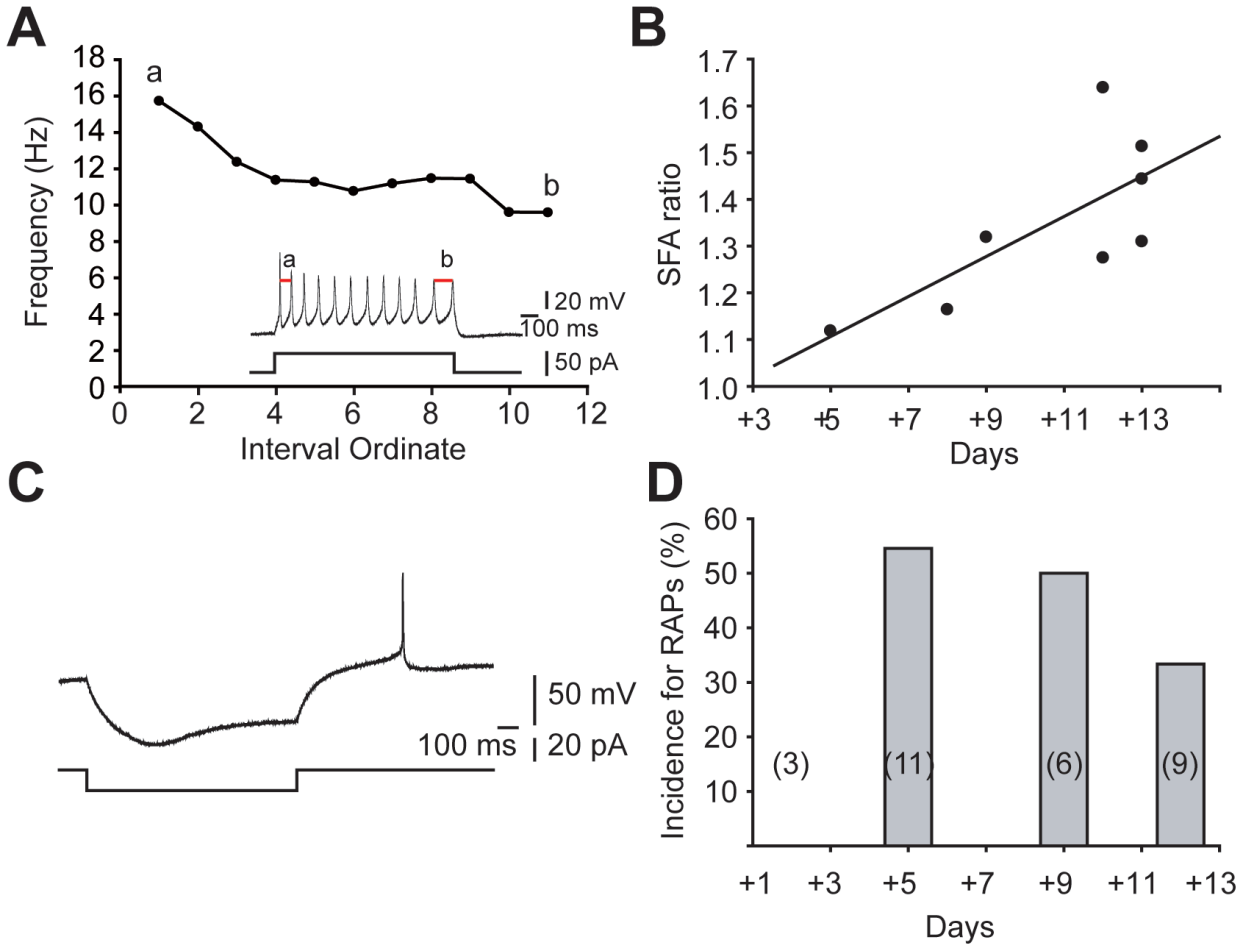
Spike frequency adaptation (SFA) and rebound action potentials (RAPs) in hESMNs. (A) An example of the change in instantaneous frequency during a train of action potentials evoked with positive current injection for 1 sec. Inset shows APs (upper) and injected currents (bottom). APs from which ‘a’ and ‘b’ ISI values were measure for SFA calculation are indicated. (B) SFA ratio, calculated as the maximum value of normalized ISIs after any amplitude of positive current injection, increased with DIV (n  = 8, R  = 0.73, P<0.05, Pearson’s linear regression). (C) RAPs were observed in a large subset of hESMNs. Upper trace shows voltage change after negative current injection. Bottom trace shows injected negative current steps. RAP follows the return of current to baseline after the hyperpolarizing step. (D) Incidence of RAPs in hESMNs at 4 different ages as indicated in Fig. 3 legend (n  = 29). Negative current steps with 5 pA increments (to at least 20 pA) were injected while checking for RAPs.

In addition to repetitive firing, we investigated whether hESMNs have other physiological properties similar to motor neurons *in vivo*. Past reports showed a time-dependent decrease in action potential discharge rate during a sustained depolarization (increase in ISI) in spinal motor neurons or SFA [10], [21], [22], [23], [24]. Figure 4A shows an example of the change in instantaneous frequency measured throughout the repetitive firing response to a depolarizing stimulus. In general, instantaneous frequency drops throughout the train. To facilitate comparison of SFA at different ages, the SFA ratio was determined by calculating the ratio of ISI measured between the last two action potentials by ISI between the first two action potentials as illustrated in the inset of Figure 4A (and see Materials and Methods). SFAs increased with days *in vitro* (Fig. 4B, n  = 8, R  = 0.73, P<0.05, Pearson’s linear regression), suggesting expression of conductances underlying SFA increased over the culture period.

In addition to SFA, we observed rebound depolarization after termination of hyperpolarizing current pulses in some hESMNs. These post-inhibitory rebound depolarizations sometimes reached threshold for triggering an action potentials or RAPs, as shown in Figure 4C. In this study, none of the hESMNs tested at 31+2 DIV exhibited RAPs even after hyperpolarizations to ∼−90 mV, although one of the neurons exhibited post-inhibitory rebound depolarization without reaching threshold for firing an action potential. However, 35 to 55% of hESMNs produced RAPs at 31+5 DIV and later (Fig. 4D). A similar age-dependent development of RAP has been observed in brainstem slices from rat hypoglossal motor neurons [15], [25]. These results suggest that as hESMNs mature morphologically, they simultaneously acquire membrane properties similar to maturing motor neurons *in vivo*.

Rebound action potentials are due to post-inhibitory rebound depolarization and this depolarization has been shown to be due to activation of inward rectifier channels, low voltage activated Ca^2+^ channels, or both [17]. Inward rectification is evident in current clamp recordings as voltage sag back towards resting potential during a hyperpolarizing current injection (Fig. 4C). Five out of 26 neurons in our study met our criteria for sag (defined in Materials and Methods). All 5 of these neurons also had RAPs, suggesting the presence of sag could contribute to the depolarizing rebound and RAPs. Conversely, approximately half of the neurons with RAP had sag (5/12), suggesting the presence of sag was not required for RAPs.

## Discussion

If hESMNs are to be useful for modeling human motor neuron development and function, it is important that they acquire mature functional characteristics similar to motor neurons *in vivo*. Here we provide evidence that hESMNs follow a time dependent course of maturation *in vitro* that can be measured by changes in morphology and electrophysiology. This maturation culminates in functional physiological properties typical of maturing motor neurons *in vitro* and *in vivo*.

### Developmental Changes in Morphology in hESMNs

Neuronal processes, including dendrites and axons, are essential elements in neural network connectivity. Mature mammalian spinal motor neurons have large cell bodies, long peripheral axons and extensive, long, branched dendrites that receive synaptic contacts across their entirety [26], [27]. From day 31+2, 2 days after plating, to day 31+5 *in vitro*, hESMNs showed a rapid increase in neuritic outgrowth and branching (Fig. 1C,E, F). Neurite outgrowth continued to increase over the subsequent 4 days (Fig. 1F). In addition, a small but significant increase in soma area was observed towards the end of the *in vitro* observation period (Fig. 1C, D). By comparison, in mouse motor neurons *in vivo*, dendritic branching is maximal between embryonic day 13 (E13) and E15 at which time it starts to decrease. Rat and human motor neurons *in vivo* also show increases in number of processes and cell body size beginning gradually at rat E14, human gestational week 5.5, and have large cell bodies with typical multipolar morphology by rat E17, human gestational week 10.5 [6]. Mouse and rat motor neurons then show little change in complexity over the first two postnatal weeks [28], [29]. The significant changes in hESMN morphology we have described, a steady increase in number of primary neurites, an increase in soma size, and an increase in total outgrowth, are consistent with the morphological changes embryonic rodent and human motor neurons display during development *in vivo.*

### Developmental Changes in Membrane Properties Expressed in Maturing hESMNs

Intrinsic membrane properties of motor neurons change with maturation, including a more hyperpolarized resting membrane potential and decreased input resistance [5], [7], [8]. Similar changes have also been reported in developing motor neurons derived from mouse ES cells (mESMNs) [12], [30]. In our studies, we have observed a decrease in input resistance but no change in resting potential. It is possible that we did not detect a change in membrane potential because we did not begin our recording until the second DIV. In studies with mESMNs, the greatest change in resting potential occurred over the first 2 DIV [12].

Physiological properties of developing rodent motor neurons change over the period of late embryonic development in a manner that is mirrored by hESMNs maturing in culture. At E15, rat motor neurons fire single action potentials with relatively long durations and shorter peak amplitudes relative to motor neurons in the postnatal rat [9]. Even E13 mouse spinal neurons grown in culture for less than 24 hours are able to fire single action potentials [31]. After birth, not only do action potentials recorded from rat motor neurons have a shorter duration and larger peak amplitude than embryonic motor neurons, they fire repetitively to a depolarizing current injection [9]. Similarly, mouse ES cell-derived motor neurons are able to fire repetitively by 3–4 DIV [12].

At early times *in vitro*, hESMNs showed several of the same characteristics as functionally immature, embryonic rodent motor neurons and mESMNs. They had single action potentials with relatively long durations even after long lasting (1 s) large depolarizing current steps. By 31+8/31+9 and 31+12/31+13 DIV, hESMNs demonstrated the ability to fire a train of action potentials in response to a sustained depolarizing current step. The first action potential in the response had a significantly shorter duration than the single action potentials recorded from hESMNs at 31+2 DIV. Thus mature hESMNs acquired comparable electrophysiological properties to mature rodent motor neurons [10]. The mechanisms underlying the developmental changes in rat motor neurons observed neonatally and mESMNs include contributions from Na^+^, K^+^, and Ca dependent K^+^ currents [9], [12]. While we did not attempt to identify specific conductances responsible for these developmental changes in hESMNs, we can speculate that similar changes may be occurring.

### hESMNs Appear to have Characteristic Active Membrane Properties of Motor Neurons

Motor neurons fire rhythmically during locomotion. Synaptic inputs onto motor neurons as well as intrinsic membrane properties of motor neurons contribute to this rhythm [32], [33]. We have demonstrated two membrane properties in hESMNs that are induced by depolarizing and hyperpolarizing current pulses injection, SFA and RAP, respectively, that contribute to the firing pattern of motor neurons.

SFA in motor neurons is modulated by activity [14] and may contribute to efficient muscle contraction [13]. Repetitive stimulation of motor nerves was used to identify the pattern most optimal to produce efficient muscle contraction by Stein and Parmiggiani (1979). The optimal pattern consisted of a short first ISI followed by longer subsequent intervals, similar to the firing pattern that results from our depolarizing current pulses injection in hESMNs referred to as SFA (Fig. 4A, B). Slow inactivation of rapidly activating sodium channels has been shown to contribute importantly to SFA in mouse motor neurons [23]. Also consistent with increased expression and importance of sodium channels in hESMNs over DIV is the decrease in action potential half width together with the lack of change of rheobase and hyperpolarization of voltage threshold. In the face of strongly decreasing input resistance, all of these properties are consistent with an increased expression and density of fast sodium channels in hESMNs. Thus it is possible that these same sodium channels contribute to the increase in SFA with maturation of hESMNs that we have observed.

Rebound action potentials have been described in many neuronal systems. It is possible that activation of an inwardly rectifying current is the main cause of RAP in motor neurons [17], although other studies have suggested the contribution of low voltage-activated Ca^2+^ currents [7], [34]. RAP may contribute to rhythmic activity of motor neurons during locomotion [7], [17], suggesting that RAP is a hallmark of functional motor neurons. Some studies have demonstrated that RAPs contribute to shaping the pattern of neuronal firing. For example, observations of motor nerve function in Xenopus have shown that RAP plays a key role in the genesis of rhythmic motor pattern [35]. Experimentally induced RAP was able to initiate or modulate the bursting discharge of rat motor neurons during fictive locomotion [17]. In our studies, hESMNs showed this firing behavior characteristic of motor neurons in response to current pulses. Moreover, those characteristic behaviors in hESMNs were age-dependent (Figs. 4D) in that RAP was not apparent at 31+2 DIV whereas it was apparent at 31+5 to 31+13 DIV. These results support the idea that hESMNs progressively develop similar conductances to motor neurons *in vivo* that underpin their physiological function.

### Variability in Morphological and Electrophysiological Properties of hESMNs

Our study revealed not only increasing maturity of electrophysiological and morphological properties of hESMNs over time in culture but also increasing variability of these parameters among the neurons tested at each time point. At least two mechanisms might contribute to this increasing variability. First, motor neuron birth is not synchronous in rodents or humans *in vivo* but follows a rostral to caudal progression (approximately embryonic day 30–50 in human) [6], [36]. Analogous ongoing motor neuron neurogenesis *in vitro* might contribute to the variability of motor neuron functional characteristics as young, immature neurons are generated. Second, motor neurons at each spinal level are developmentally diversified into multiple subtypes [37], [38], exhibiting diverse morphology and connectivity [39], [40]. The variability of membrane properties we observed may reflect this variety of motor neuron subtypes. For example, it has been shown that back-labeled, embryonic chick motor neurons from the medial and lateral motor columns had significantly different input resistance and whole cell capacitance [41]. Deliberate specification of diverse motor neuron subtypes from stem cells has recently been demonstrated [42], [43]. It will be interesting to determine in future experiments whether the diversity in electrophysiological and morphological properties observed *in vitro* can be attributed to molecularly defined populations of motor neurons.

Maturation of hESMNs *in vitro* followed a time course surprisingly similar to motor neuron maturation in late rodent embryonic development. As action potentials recorded from hESMNs themselves became faster and more robust, the pattern of firing mimicked that of newborn rodent motor neurons. While it is not possible to study human motor neuron maturation *in vivo* with this same resolution, hESMNs provide not only a model system in which function and development of human motor neurons can be studied, but also may provide a way to investigate human motor neuron diseases for which iPS-MNs are now becoming available.

## Materials and Methods

### Ethics Statement

The work performed in this paper on motor neurons derived from human embryonic stem cells has been approved by Columbia University ESCRO committee (Embryonic Stem Cell Research Oversight committee).

### ESC Culture

A human embryonic stem cell line with a motor neuron reporter (BAC-*Hb9*::GFP) [19] was grown under standard pluripotency maintenance conditions: on irradiated CF-1 mouse embryonic fibroblast feeder cells (0.015 M cells/cm^2^, GlobalStem) seeded on gelatinized (Millipore) tissue culture plastic. Cells were fed daily with ES Medium comprised of Dulbecco’s Modified Eagle Medium: nutrient mixture F-12 (DMEM:F12, Invitrogen) with 20% Knockout Serum Replacer (Invitrogen), 110 µM beta-mercaptoethanol (BME; Sigma), L-Glutamine and Non Essential Amino Acids (NEAA; Invitrogen), and 20 ng/ml basic fibroblast growth factor (bFGF; Invitrogen) (ESC medium), and passaged weekly using 50 µg/ml dispase for 20 min (Invitrogen) followed by manual trituration. Parallel passages of ESCs were karyotyped at subsequent passages and found to be normal.

### Motor Neuron Differentiation

After normal passage, washed ESC colonies were incubated for 1 hour in ESC medium, as described above, with 10 µM Rho-associated kinase (ROCK) inhibitor (Y-27632, Ascent Scientific) [20], then trypsinized to single cells and seeded in suspension at 0.4 M cells/ml in ES medium with 10 µM ROCK inhibitor, and 300 ng/ml recombinant mouse Noggin (R&D). Fresh ROCK inhibitor, FGF, and Noggin were added daily for the first 6 days. Embryoid bodies (EBs) were pelleted at 100 G on day 4 and resuspended in DMEM F:12 plus N2 supplement (Invitrogen), NEAA, L-Glutamine, 2 µg/ml Heparin (Sigma), bFGF, Noggin, and ROCK inhibitor. EBs were pelleted and fed with fresh medium every other day until day 31. ROCK inhibitor was last added at day 5. Noggin and bFGF were discontinued at day 10, and 1∶10 dilution of Wnt3a-L-cell conditioned medium (ATCC), all-trans retinoic acid (RA, 100 nM, Sigma), ascorbic acid (0.4 µg/ml, Sigma), db-cAMP (1 µM, Sigma), and recombinant mouse Sonic hedgehog (SHH) protein (100 ng/ml SHH-C25II, R&D) were added from day 10 onward. On Day 18 Wnt3a-conditioned medium was discontinued, SHH was increased to 200 ng/ml and recombinant human brain-derived neurotrophic factor (BDNF; 10 ng/ml, R&D) was added. At day 25, base medium was switched to Neurobasal with N2 and B27 (Invitrogen), L-Glutamine, NEAA, ascorbic acid, db-cAMP, (Neural Differentiation Medium (NDM)), with 10 ng/ml each recombinant human BDNF, glial cell-derived neurotrophic factor (GDNF), insulin-like growth faction 1 (IGF-1), and ciliary neurotropic factor (R&D), 200 ng/ml SHH, and 100 nM RA.

EBs were dissociated using trypsin on day 31 and cryopreserved using EmbryoMax 2× freezing medium (Millipore) for future use. Separate vials were thawed for electrophysiology and morphology time series, seeded on poly-ornithine/laminin coated glass coverslips, in complete day 25 NDM including all supplements. However, RA was reduced to 10 nM and SHH was reduced to 20 ng/ml and the following supplements were added: 1 µg/ml mouse laminin (Invitrogen), BME (25 µM, Sigma), glutamate (25 µM, Sigma), forskolin (20 µM, Sigma), and IBMX (100 µM, Fisher) at 250 K cells per 35 mm coverslip or 46 K cells per 15 mm coverslip. Half the medium was changed every 4 days.

### Immunocytochemistry

Cultures were fixed for 30 minutes in 4% paraformaldehyde (PFA) in phosphate buffered saline (PBS) at 4°C, washed 3 times for 5 min in PBS, quenched and permeabilized in wash PBS plus 0.1% Triton X-100 (Wash buffer) plus 50 mM glycine for 15 min. Samples were blocked with Wash buffer plus 10% normal donkey serum for one hour, incubated with primary antibody in blocking buffer (chicken anti-GFP 1∶1000, Invitrogen A10262; mouse anti-HB9,1∶50, MNR2/815C10-s, Developmental Studies Hybridoma Bank) overnight. Cells were then washed, incubated with DyLight coupled donkey anti primary-species IgG antibodies (Jackson Immunoresearch, 1∶1000). Finally, cells were washed and counterstained with DAPI (Invitrogen).

### Imaging and Image Analysis

Coverslips were mounted in Fluoromount G and imaged on an inverted Zeiss AxioObserver Z1 using a 20× Plan-APOCHROMAT 0.8 NA objective and a 14-bit, gray scale Photometrics HQ2 CCD camera. Images were exported as 16 bit gray scale images for analysis. Cells were scored as HB9^+^ or GFP^+^ based on having >10 K mean gray levels of HB9^−^ or >40 K mean gray levels of GFP-channel fluorescence intensity over local background, respectively, using the Metamorph Multiwavelength Cell Scoring module. Neurites were traced using the Neurite Outgrowth module (Metamorph) based on GFP fluorescence intensity of >3,000 g.l. over local background. A 20× field of view was manually adjusted to capture the maximum neurite outgrowth for each cell. This field size was sufficient to capture all neurites for almost all cells. At day 36 for example, the day at which the peak of total median outgrowth per cell occurs, less than 9% of GFP^+^ cells had neurites extending beyond the field of view. Morphological measurements were grouped by days in culture and SigmaPlot11 was used for statistical analysis.

### Electrophysiology

Whole-cell patch recordings were made from *Hb9*::GFP^+^ motor neurons. Coverslips were transferred to the stage of a TE2000-E microscope (Nikon) and continuously perfused at a low flow rate of 1 ml/min with bath recording solution containing (in mM): 145 NaCl, 5 KCl, 2 CaCl_2_, 10 HEPES, 2 MgCl_2_ and 5.5 glucose, pH adjusted to 7.3 with NaOH, osmolality 325 mOsmol kg^−1^. Patch pipettes with a resistance of 3–5 MΩ were pulled from borosilicate glass capillaries (0.86 mm ID, 1.5 mm OD) using a P-97 pipette puller (Sutter Instrument Co). Intracellular solution had the following composition (in mM): 120 potassium methanesulfonate, 10 NaCl, 10 EGTA, 1 CaCl_2_, 10 HEPES, 0.5 NaGTP, 5 MgATP, 0.1% biocytin, pH adjusted to 7.2 with KOH, osmolality 280 mOsmol kg^−1^. Some of the electrophysiologically recorded cells were filled with biocytin during recording, then were fixed, stained and imaged as above. Junction potential was corrected before recording.

Data were acquired using an Axopatch 200 B amplifier and pClamp 10 software (Molecular Devices, Sunnyvale, CA, USA). Data were filtered at 2 kHz and digitized at 20 kHz. Action potentials were evoked by injecting depolarizing currents of 1 s duration and analyzed using AxoGraph X software (AxoGraph Scientific, Sydney, Australia). Action potential characteristics in hESMNs were measured from resting membrane potential. The criterion for identification of a first action potential was when a voltage response to depolarizing current injection had obvious threshold, visible as a rapidly rising membrane potential that was positive to 0 mV. Rheobase was defined as the minimum current step amplitude required to evoke an action potential. Voltage threshold for action potentials was measured as in [44]. Action potential half-width was measured at rheobase. In a given cell, ISIs were calculated from recordings in which the maximum number of action potentials were evoked. Spike frequency adaptation (SFA) ratio was calculated as: ISI_last_/ISI_1st_. The calculation was performed only on data from neurons in which more than 5 action potentials could be evoked by a 1 s depolarizing current step. Sag was analyzed as the response following a hyperpolarizing current step that fit the following criteria: 1)The ratio between the size of voltage change at the steady state and the peak during a 1 sec current injection was greater than 1.1 and 2) the voltage change peaked during the first half of the current injection period.
